# APOBECs: Our fickle friends?

**DOI:** 10.1371/journal.ppat.1011364

**Published:** 2023-05-18

**Authors:** Jaquelin P. Dudley

**Affiliations:** Department of Molecular Biosciences and LaMontagne Center for Infectious Disease, The University of Texas at Austin, Austin, Texas, United States of America; University of Iowa, UNITED STATES

## How do APOBECs function as antiviral factors?

The apolipoprotein B mRNA-editing catalytic polypeptide-like (APOBEC) family specifies multiple cytidine deaminases. In mammals, a minimum of 5 genes encode APOBECs 1, 2, 3, 4, and activation-induced cytidine deaminase (AID) [1,2]. The A1 enzyme was the first to be recognized and plays a critical role in tissue-specific editing of particular host mRNAs [3], but a confirmed role in mutagenesis of viral genes has not been established. Humans have amplified the APOBEC3 (A3) locus to yield 7 members: A3A, A3B, A3C, A3D, A3F, A3G, and A3H. All of the APOBEC proteins appear to bind to single-stranded RNA or DNA or both [1,2]. APOBEC enzymes deaminate single-stranded nucleic acids on cytosines, leading to C-to-U mutations. When these mutations occur on the minus strand of replicating viruses, the result is a G-to-A transition on the viral plus strand. Since these transitions often lead to nonsense or missense mutations, synthesis of essential viral gene products is blocked and infectious particle yields decline [1,2]. In response, viruses make multiple genes that interfere with the function of A3 proteins. Generally, these are protein antagonists, including the well-known example of HIV-1 Vif, which acts as an adapter to an E3 ligase to induce proteasomal degradation of some A3 deaminases [4–7]. This example provides clear evidence that the function of the A3 genes is to interfere with viral replication. A3s appear to be expressed in higher amounts in lymphoid and myeloid cells [8–10], suggesting that these enzymes act as frontline defenders from viral invasion. However, other cell types, such as mammary cells, also express A3s [11]. Since some A3s are packaged into viral particles, virion incorporation of A3s provides additional protection for newborns who ingest milk-transmitted viruses.

Although deamination of retroviral DNA is likely the primary mechanism of viral inhibition, deamination-independent modes of APOBEC activity have been observed [12,13]. Because A3s bind to single-stranded RNA packaged into virions, these deaminases provide a road block to viral DNA synthesis [13–15]. A3G also interacts with HIV-1 reverse transcriptase (RT) to interfere with DNA replication [16]. Different APOBECs may have evolved to allow binding to retroviral enzymes other than RT. A3 enzymes also have been shown to inhibit DNA and RNA-containing viruses, such as human papilloma viruses and coronaviruses, respectively [13–15]. APOBEC binding to other viral polymerases would allow ample opportunities for blocking replication of diverse viruses.

## Are there multiple mechanisms for viral Apobec antagonists?

The betaretrovirus mouse mammary tumor virus (MMTV) has provided a model for the first in vivo studies of APOBEC3 activity [17]. MMTV requires replication in B and T lymphocytes [18,19]. These cells serve as reservoirs for infection of mammary epithelial cells, which produce virions for transmission through milk [20]. Previous results have shown that MMTV replication was enhanced in murine A3 (mA3)-knockout mice on the C57BL/6 (B6) background [17]. Although mA3 was packaged into virions in wild-type B6 mice, MMTV DNA hypermutation was not detected, suggesting a deamination-independent mechanism of viral inhibition. Nevertheless, no viral antagonist of mA3 was identified [17]. The Rem protein has been identified as an MMTV-encoded inhibitor of AID, an APOBEC enzyme that is primarily expressed in B lymphocytes during differentiation to antibody-producing cells [21]. AID also is suppressed by an Epstein–Barr virus-encoded microRNA (miR) [22], suggesting that AID targets viruses that infect B cells. Interestingly, although AID has been shown to function in class switch recombination and somatic hypermutation of immunoglobulin genes, it likely evolved to counteract retrotransposition of long interspersed nuclear elements (LINEs) [23], which occur in multiple cell types.

WRC (W = A/T, R = A/G, C) motifs in MMTV proviruses that are unable to produce Rem are heavily mutated in BALB/c mice to produce stop codons compared to proviruses that express Rem [21]. This difference disappeared after infection of *Aicda^-/-^* mice with Rem-mutant MMTV. Infection of *Aicda*^*-/-*^ mice greatly reduced proviral mutations in WRC motifs, which are known AID-targeting sequences in nuclear immunoglobulin genes [24]. In contrast, AID is reported to inhibit LINE-1 elements through a deaminase-independent mechanism [25]. The mA3 TYC-target motifs (T, Y = C/T, C) in MMTV proviruses observed in BALB/c mice also increased in the absence of Rem but, surprisingly, the mA3-type mutations in Rem-null proviruses disappeared when AID was absent. Furthermore, coexpression of Rem in tissue culture cells with either AID or mA3 resulted in proteasomal degradation of AID, but not mA3 [21]. These results suggested that Rem has HIV-1 Vif-like properties, yet inhibits AID and mA3 differently. One possibility is that AID stabilizes mA3 levels indirectly through a complex with viral RNA and AID. The Bet proteins of spumaretroviruses apparently use protein–protein interactions for binding to A3G, thus preventing deaminase packaging into virions [26]. MMTV Rem does not block mA3 packaging [21], consistent with another mode of action. Nevertheless, the idea that 1 APOBEC protein influences the activity of additional deaminases is intriguing.

In contrast to other APOBEC enzymes, the roles of A2 and A4 proteins remain mysterious. Recent data suggest that these deaminases may have evolved from enzymatic activities to sole use of their nucleic acid-binding properties. Recent work has hinted that A2 and A4 bind to promoter regions to affect transcriptional activity of selected genes [27]. Alternatively, by analogy to the ability of human A3G to bind RT [16], it is possible that some APOBEC proteins bind to host or viral transcription factors or their regulators, allowing modulation of their activity.

## Do APOBECs contribute to viral evolution?

The answer most certainly is affirmative. Ample evidence indicates that replication of viruses in APOBEC-expressing cells has led to the appearance of novel viral variants [28]. As noted previously, although some variants are nonviable, viruses also make APOBEC antagonists. One may also speculate that allocation of valuable viral genetic resources to inhibit APOBECs is a measure of how effectively these enzymes block virion production. Application of Vif inhibitors as therapeutics has resulted in the development of drug-resistant HIV-1 variants that are more efficient at replication in the presence of human A3G than wild-type virus [29]. Since viral antagonists are only partially successful, the immune system selects for replication-competent survivors, leading to a virus–host “arms race” [30]. Studies in HIV-1-infected patients reveal that increased APOBEC expression promotes disease progression, whereas the ability of the major histocompatibility complex (MHC) class I proteins to generate virus-specific cytotoxic T cells leads to viral control [2].

In addition to the known effects of APOBECs on HIV-1 variation acting at the level of DNA replication [16], the Coronavirus Disease 2019 (COVID-19) pandemic has provided a current stage for the observation of RNA-containing virus evolution. More than 65% of the recorded mutations in the Severe Acute Respiratory Syndrome Coronavirus 2 (SARS-CoV-2) genome are estimated to result from interactions with APOBECs and adenosine deaminase acting on RNA (ADAR) [31]. Surveys of hundreds of CoV-2 genomes revealed that C-to-U mutations are approximately 6-fold more common than U-to-C mutations when compared to the Wuhan-Hu-1 strain. In contrast, this mutation bias was not observed for Ebola isolates [25]. These data argue that APOBECs induce mutations that are a source of selection and viral evolution.

**Fig 1:**
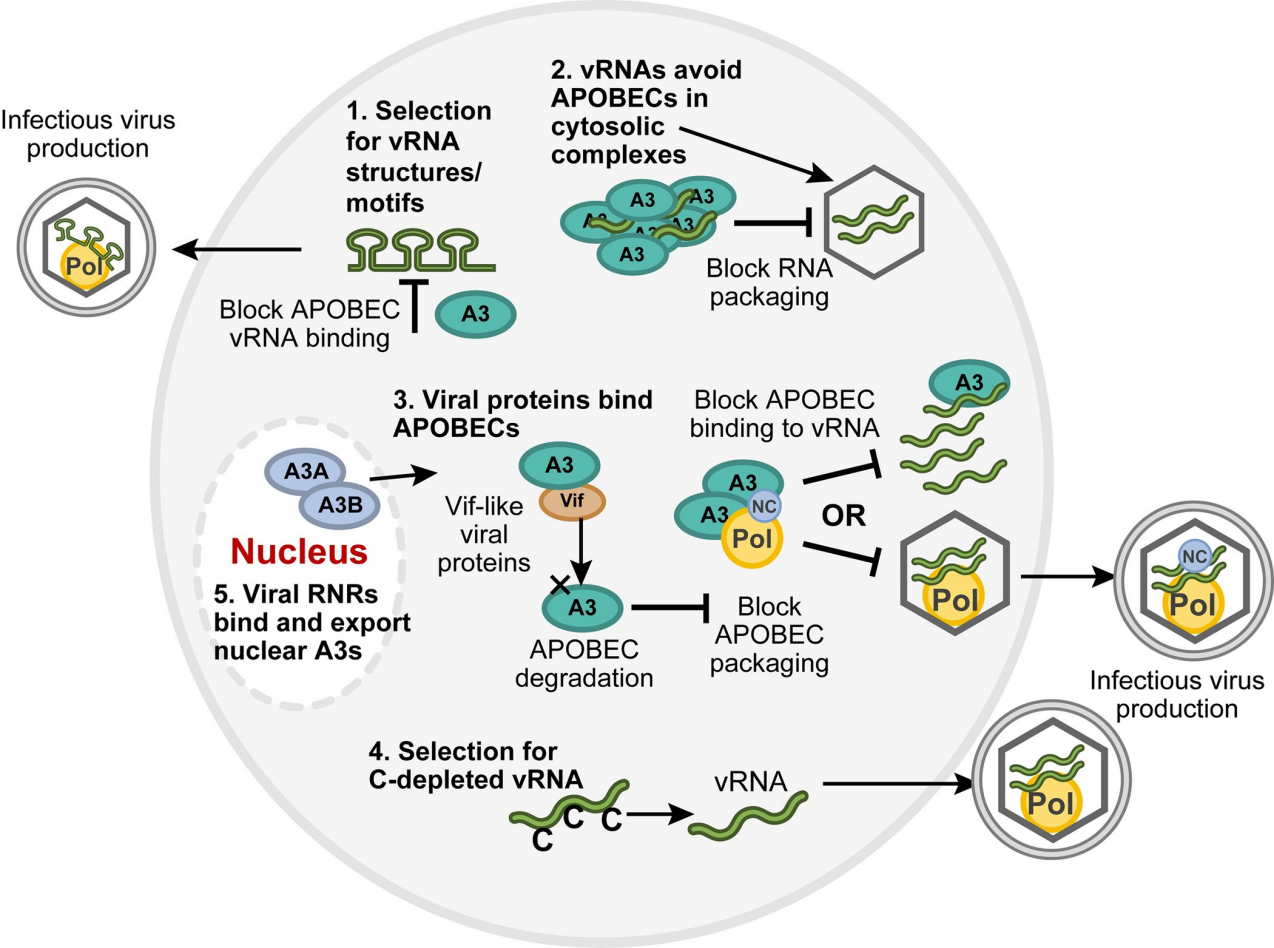
Potential mechanisms for virus resistance to APOBEC-mediated inhibition. (1) vRNAs selected for the absence of certain sequence motifs prevent APOBEC binding or mutagenesis. (2) Viral genomes may avoid repressive complexes with APOBECs (e.g., A3) that interfere with RNA packaging into virions. (3) Virion proteins that bind to APOBECs may have different consequences including A3 degradation or exclusion from virions. Viral proteins, such as MMTV Rem or HIV-1 Vif, lead to proteasomal degradation of their targeted APOBEC proteins. A3 binding to NC, an abundant viral protein, or foamy virus Bet prevents A3 packaging into virus particles. Virion exclusion of A3 will avoid inhibition of viral replication in target cells. A3 in virions also may be inhibited by binding to polymerases (Pol), such as RT. (4) Selection of viral RNA genomes that have few cytidine residues will provide limited opportunities for APOBEC-mediated mutations. (5) RNRs encoded by gammaherpesviruses, DNA viruses that replicate in the nucleus, block deaminase activity and/or promote the export of A3A and A3B enzymes to the cytoplasm. APOBEC, apolipoprotein B mRNA-editing catalytic polypeptide-like; MMTV, mouse mammary tumor virus; NC, nucleocapsid; RNR, ribonucleotide reductase; RT, reverse transcriptase; vRNA, viral genomic RNA.

Although SARS-CoV-2 and Ebola are positive and negative sense RNA-containing viruses, respectively, both have been implicated as cross-species transmission events to humans, perhaps from bats [25]. Bats are known to have an extensive array of APOBEC genes, which may result in both tolerance for viral infection and a source of variability. Moreover, bats recently have been shown to duplicate the gene encoding PKR, suggesting that these animals are primed to induce interferons (IFNs) and innate immune responses [32]. Why then are some viruses susceptible to APOBEC-mediated mutation, whereas others are not? Several explanations for viral resistance to the effects of APOBECs are possible (Fig 1). (i) Mutations occur preferentially in both primary sequence and secondary structural contexts [33]. This observation suggests that some genomes will be more susceptible than others to APOBEC-mediated mutations. (ii) Another possibility is that some RNA viruses exclude APOBECs from their replication complexes. Human A3G, which is known to be a major restriction factor for HIV-1, has been reported to localize in cytosolic processing (P) bodies. P bodies and stress granules are repositories for mRNAs and translation factors [34], suggesting that APOBECs may sequester viral genomic RNA (vRNA) into specific cellular ribonucleoprotein complexes. (iii) The ability of APOBECs to bind to viral proteins, such as polymerase or nucleocapsid, may prevent viral RNA replication [2]. CoV-2 nucleocapsid protein expressed from a chimeric HIV-1 genome has been shown to associate with human A3G [25], consistent with a mechanism for virus particle incorporation independent of genomic RNA binding. Incorporation of APOBECs into viral particles is not required for viral inhibition since exclusion of AID from MMTV virions does not prevent proviral mutagenesis [21]. (iv) Reduction of cytidine residues (and target motifs) from the viral RNA genome decreases susceptibility to APOBEC-induced mutations [25]. (v) Some gammaherpesvirus proteins trigger the export of APOBEC enzymes that function in the nucleus [35,36]. Although this list is not comprehensive, more than 1 mechanism likely provides susceptibility or resistance to APOBEC-mediated inhibition of individual viruses.

## How does innate immune signaling regulate APOBEC activity?

The regulation of APOBEC expression is only partly understood. APOBEC cytidine deaminases are part of the innate immune system, yet multiple reports indicate that other functions of innate immunity, such as signaling through the type I IFNs and Toll-like receptors (TLRs), increase levels of the A3 enzymes [37–39]. The induction of APOBECs occurs primarily at the transcriptional level. The 7 human A3 enzymes are differentially expressed in different cell types and stages of differentiation [40]. However, induction of the hA3 enzymes has been documented in multiple myeloid and lymphocyte lineages, which quickly encounter invading pathogens [8]. Recent work has suggested that human A3A competes for binding to IFN-stimulated regulatory elements (ISREs) found upstream of a subset of genes, perhaps providing a feedback loop to dampen innate immune responses after initial pathogen encounters [41]. Therefore, although APOBECs provide friendly antiviral functions, their activity appears to be deleterious if unregulated, similar to other immune responses.

Regulation of AID expression reveals the multiple pathways that control APOBEC transcript levels. Treatment of cultured B cells with lipopolysaccharides (LPS) (TLR-4 signaling) in conjunction with the cytokine interleukin-4 (IL-4) is known to induce AID [42]. In B cells, IL-4 signals through the IL-4 type I receptor to activate the signal transducer and activator of transcription 6 (STAT6) protein [43]. Thus, both TLR and cytokine signaling result in activation of NFkB and STAT transcription factors (as well as others) to increase RNA levels of downstream targets, including the genes encoding AID, IFNs, and cytokines [42]. Pathogen infections also increase AID mRNA levels through B cell receptor (BCR) and CD40 ligand receptor (CD40L) signaling during adaptive immune responses [44,45]. Some pathways are activated in infected cells outside of the hematopoietic lineage to induce AID with mutagenic consequences for both pathogens and host [42].

## APOBECs: Our fickle friends?

Evolutionary evidence suggests that APOBECs are advantageous because of the expansion of this gene family in higher organisms and the amplification of the A3 genes in primates. Many of these genes appear to be under positive selection [1,30]. The duplication of deaminase domains in some APOBECs suggests additional selection for function. APOBEC function has expanded from AID control of retrotransposons in innate immunity to the evolution of the adaptive immune system with antibody affinity maturation [23]. Additional contributions to immune function may be achieved using RNA editing by A3A in monocytes and macrophages [46]. Despite such advantages, substantial evidence indicates that APOBECs have their downsides. APOBEC-mediated mutagenesis of pathogens results in the emergence of immune and antiviral drug escape variants, which have been observed during the HIV-1 and COVID-19 pandemics [28,47]. Recent evidence from studies of gammaherpesviruses indicates that some primates have lost A3B, which is a nuclear enzyme like A3A, suggesting that this enzyme may have deleterious consequences for its host [35]. Therefore, APOBECs allow immune diversification while driving adaptation by microbes.

Although previous reports have suggested that viruses with DNA replication intermediates are the most affected, more recent experiments argue that RNA-containing viruses, such as rubella (*Matonaviridae*) and SARS-CoV-2 (*Coronaviridae*), have C-to-U editing typical of APOBECs [15,48]. Rubella virus quasi-species were isolated from the granulomas of children with primary immunodeficiencies after immunization with the RA27/3 vaccine strain. These variants were infectious and revealed evidence of C-to-U editing, primarily on the positive strand and enriched for UC motifs typical of APOBECs. This study also indicated that the vaccine-derived rubella quasi-species were recovered from the nasal cavity, but not in the bloodstream, suggesting that APOBEC-induced mutations could serve as a source of novel viruses for respiratory transmission [48]. The quasi-species showed decreased cytopathic effects compared to the vaccine strain, which presumably contributed to their persistence.

APOBEC activity against coronaviruses also has been reported [15,28,49]. These studies report the appearance of C-to-U mutations similar to rubella virus [15]. Overexpression of A3A, but not A1 or A3G, induced mutations during SARS-CoV-2 replication in cultured colon epithelial cells (Caco-2) that express ACE-2, the CoV-2 receptor [50,51]. Unexpectedly, such mutations, which occurred in the CoV-2 5′ untranslated region, appeared to confer a replication advantage and have been isolated as variants in the human population [28]. A more recent study also observed C-to-U mutations in SARS-CoV-2 viruses infecting 293T cells transiently transfected with APOBEC expression plasmids [49]. Only expression of A3A, but not a catalytic site mutant, revealed C-to-U mutations. Infection of Calu-3 (lung carcinoma) cells with SARS-CoV-2 treated with IFNβ and TNFα to induce A3A increased C-to-U mutations, which was eliminated in A3A-knockout cells [49]. Nevertheless, the number of mutations observed in culture was low. Although further evidence for APOBEC editing of SARS-CoV-2 is needed, both in vivo and in cell culture systems suggest that RNA-containing viruses, in addition to retroviruses, have edited genomes typical of APOBECs. These mutations lead to persistence of viral infections as well as novel variants that evade immune responses, consistent with the idea that APOBECs are our foes.

Nonetheless, multiple mechanisms used by APOBECs likely take a heavy toll on viral infections, increasing the likelihood that an inapparent infection with low transmission will result. In addition, APOBECs have been reported to induce mutations in specific cellular genes, particularly those expressed in macrophages and monocytes [46]. These data indicated that multiple cellular genes, including those activated by hypoxia, IFNs, and macrophage differentiation, were subjected to RNA editing. Many of the C-to-U changes were in coding regions and caused nonsynonymous changes [46,52]. Similar to rubella virus, the mutations were present in the loop of a stem-loop region. Although the ability of APOBECs, other than A1, to induce mutations in RNA has been controversial, these experiments showed that A3A had the highest level of induction in macrophages compared to other APOBECs. Knockdown also reduced C-to-U mutations in host target RNAs [46]. Although functional effects were not demonstrated in this paper, more recent data suggest that RNA editing may affect M0 to M1 macrophage polarization and be induced by viral infection [53].

A similar “Janus” (the two-faced god) scenario emerges for the role of APOBECs in human cancers, of which approximately 10% are due to viral infections [54]. Interestingly, repeat elements, such as retrovirus-like LINE-1s, are activated in human colon carcinomas. These colon cancers, particularly those lacking the tumor suppressor p53, showed decreased invasion when treated with the RT inhibitor 3TC. Treatment with 3TC increased production of innate immune proteins, including A3B. Knockdown of the cytosolic DNA sensor STING partially reversed the 3TC effect on tumor cell migration, which was attributed to detection of RT intermediates [55]. However, APOBEC proteins also might serve as DNA sensors. Guo and colleagues noted that distinct APOBEC family members (A3C, A3D, A3F, and A3H) were often coexpressed, perhaps defining specific immune populations in the tumor microenvironment. High tumor infiltration with immune cells was correlated with a better patient prognosis [56]. Thus, APOBEC expression in tumor cells as well as surrounding immune cells likely affects cancer patient outcomes.

Current databases have designated nuclear APOBECs, such as A3A, A3B, and AID, as relevant to human cancer [56]. A3A has been annotated as an oncogene, whereas A3B has been designated as both an oncogene and a tumor suppressor gene [57]. The latter designation is consistent with the role of A3B as both friend and foe in particular tumor contexts. In this regard, AID has been annotated as both an oncogene and a “driver” gene [56]. Driver genes contribute to cancer growth in specific microenvironments [58]. AID activity is required for chromosomal translocations between immunoglobulin heavy chain and the oncogene c-*myc* [59]. In addition, the cytosolic A3G protein was considered to be a driver [56], perhaps by altering RNAs in natural killer cells [60]. Recently, a model of carcinogen-induced bladder cancer in C57BL/6 mice revealed that human A3G overexpression led to tumor acceleration in the absence of the mouse A3 gene. Surprisingly, A3G was localized to the nucleus in mice and in human bladder tumor lines [61]. These data suggest that APOBECs often function in cancer progression either directly in tumor cells or the microenvironment.

In summary, APOBECs induce mutations in both viral and host DNA, but it appears increasingly likely that RNA-containing viruses are mutated by cytidine deaminases. These mutations limit viral replication and transmission. APOBEC-mediated changes on host mRNAs in innate immune cells may provide additional short-term adaptations to inhibit viral infections. Nevertheless, these mechanisms yield viral selection. APOBEC expression in cancers leads to DNA damage and tumor cell death, yet allows for immune escape variants. Indeed, APOBECs are fickle friends.
